# A treatment within sight: challenges in the development of stem cell-derived photoreceptor therapies for retinal degenerative diseases

**DOI:** 10.3389/frtra.2023.1130086

**Published:** 2023-09-29

**Authors:** Davinia Beaver, Ioannis Jason Limnios

**Affiliations:** Clem Jones Centre for Regenerative Medicine, Bond University, Gold Coast, QL, Australia

**Keywords:** retinal degeneration, regenerative medicine, human pluripotent stem cells, photoreceptors, cell therapy, retinal organoids

## Abstract

Stem cell therapies can potentially treat various retinal degenerative diseases, including age-related macular degeneration (AMD) and inherited retinal diseases like retinitis pigmentosa. For these diseases, transplanted cells may include stem cell-derived retinal pigmented epithelial (RPE) cells, photoreceptors, or a combination of both. Although stem cell-derived RPE cells have progressed to human clinical trials, therapies using photoreceptors and other retinal cell types are lagging. In this review, we discuss the potential use of human pluripotent stem cell (hPSC)-derived photoreceptors for the treatment of retinal degeneration and highlight the progress and challenges for their efficient production and clinical application in regenerative medicine.

## 1. Introduction

The global burden of vision loss is significant, with an estimated 61 million people predicted to be blind and another 474 million people experiencing moderate to severe vision impairment by 2050 (1). The root cause of irreparable vision loss is often the degeneration of photoreceptor cells in the retina, which can occur as a primary or secondary disease process. Therefore, developing effective therapeutic approaches for retinal degenerative diseases is crucial in the fight against vision loss.

The retina is a layer of nervous tissue in the eye responsible for vision. The outer nuclear layer of the human retina contains light-sensitive cells called photoreceptors that convert light signals into electrical impulses (2). The inner nuclear retinal layer consists of bipolar, horizontal and amacrine cells that receive and modulate visual information before synapsing with the ganglion cell layer to transmit electrical impulses sent to the brain for visual processing (3).

The human retina contains approximately 110 million rods and 4.6 million cones (4). Rods account for 95% of photoreceptor neurons and are concentrated in the peripheral retina, largely absent in the fovea. They are specialized cells that provide scotopic vision, having low contrast sensitivity and acuity. Rods are extremely sensitive to a single photon of light and can become light-saturated in bright light (5).

Cones constitute 5% of photoreceptors in humans. The macula is a small region of the retina that contains the highest density of cones, resulting in high visual acuity. Humans and some primates have three cone subtypes (blue, green, red) for trichromatic vision (6). Unlike rods, cones are not light-saturated and can recover their membrane current from photobleaching within milliseconds (7).

The health and function of photoreceptors are carefully maintained within the retinal ecosystem. Retinal pigment epithelial (RPE) cells interdigitate with photoreceptors and perform critical functions, such as the phagocytosis of photoreceptor outer segments, recycling of opsins (visual cycle), barrier function, nutrient supply, waste removal, and cytokine secretion to preserve retinal function (8). Müller glial cells of the retina provide structural support and help balance neurotransmitters, trophic factors, and metabolites (9). Cones also depend on rods for their survival through the regulation of glucose uptake (10). Disturbance of the retinal ecosystem can cascade toward retinal degeneration when cell functions are compromised.

**Figure 1:**
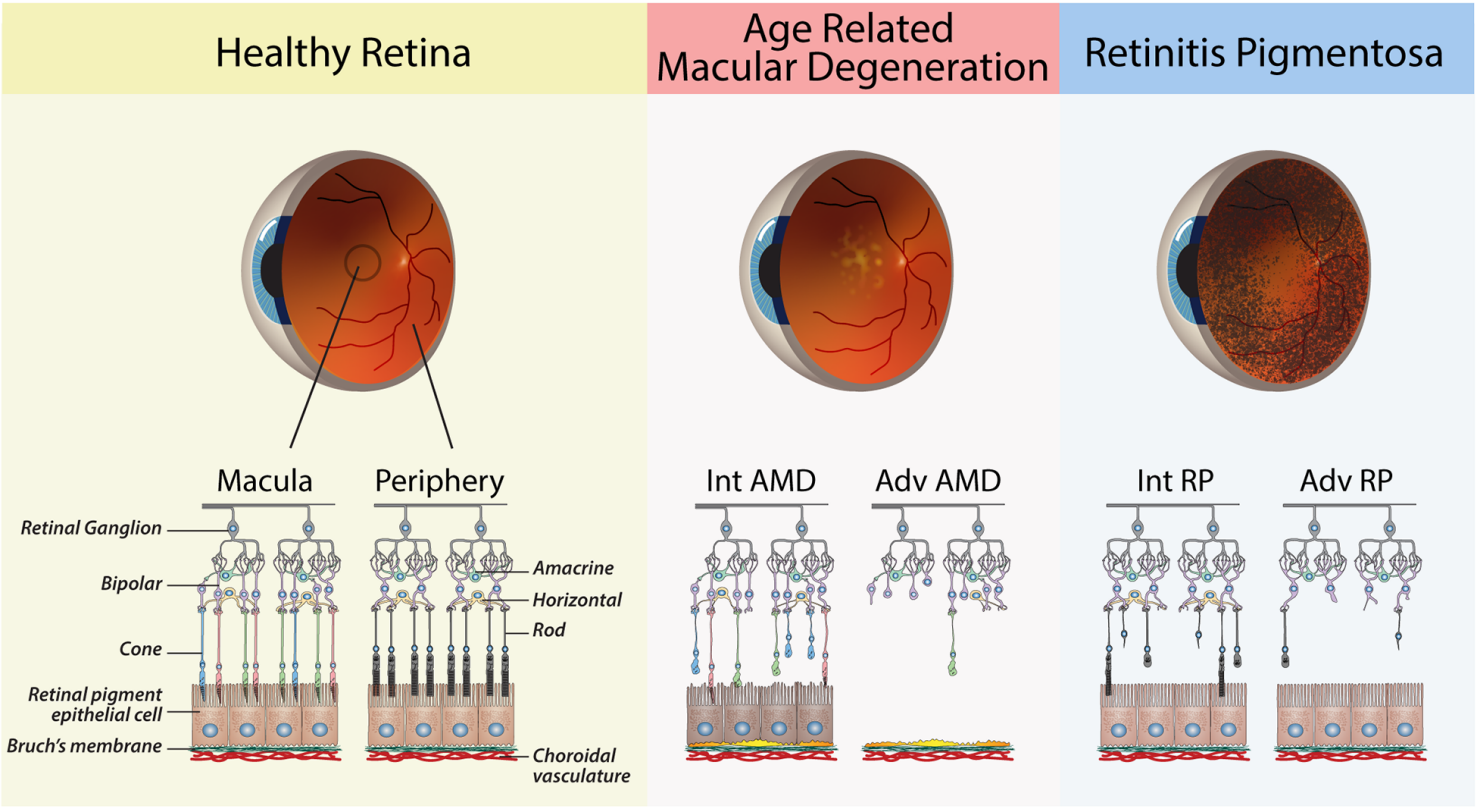
Schematic diagrams of retinas from healthy and diseased eyes. The healthy retina shows the major cells of the retina, with a clear distinction between the photoreceptors of the macula vs. the peripheral retina. Age-related Macular Degeneration: Retinal schematic of the macula, as shown by the presence of cone photoreceptors. Intermediate (Int) AMD—The presence of sub-RPE drusen deposits and early signs of RPE dysfunction. Advanced (Adv) AMD—loss of RPE cells and cones in the macula. Retinitis Pigmentosa: Retinal schematic showing the progressive degeneration of rods in the peripheral retina. Intermediate (Int) RP—partial loss of rod. Advanced (Adv) RP—widespread loss of rods in the peripheral retina.

Age-related macular degeneration (AMD) is a leading cause of blindness in elderly individuals in Western nations, affecting approximately 200 million people worldwide (11, 12). AMD begins as yellowish deposits called drusen in the retina of the macula and can progress to either dry AMD (geographic atrophy) or wet AMD (choroidal neovascularization). Late-stage AMD leads to severe vision impairment and irreversible loss of central vision due to the death or dysfunction of the RPE in the macula, followed by secondary loss of cone photoreceptors (13). Multiple contributing factors to AMD, including age, light exposure, genetics and lifestyle, lead to dysregulation of metabolic, redox and complement systems (14). While current treatments can slow the progression of AMD, approximately 15% of patients will ultimately lose their central vision (15) (Figure 1).

Inherited retinal diseases (IRDs) are a group of genetic disorders that result in blindness due to the primary death or dysfunction of photoreceptors. There are over 300 known mutations that can cause IRDs, resulting in conditions such as retinitis pigmentosa (RP), Stargardt disease (SD), cone-rod dystrophy (COD) and Leber congenital amaurosis (LCA) (16). RP is the most common type of inherited retinal disease, affecting approximately 1 in 5,000 births, and typically causes progressive vision loss beginning in the third or fourth decade of life due to the loss of rod photoreceptors in the peripheral retina (17) (Figure 1). This leads to night blindness and narrowing of vision, and ultimately complete blindness due to secondary loss of cones (18).

Current treatment options for AMD and IRDs are limited and primarily focused on slowing the progression of degeneration (19, 20). The standard treatment for neovascular AMD is anti-VEGF therapy to reduce abnormal blood vessel growth and leakage (21). In early 2023 the FDA approved Pegcetacoplan (marketed as Syfovre) as the first treatment for geographic atrophy (GA). Syfovre works by inhibiting complement factor 3 (22) and can reduce the growth of geographic atrophic regions by up to 30% compared to controls (23, 24). In addition to limited efficacy, patients with dry AMD are at increased risk of developing wet AMD (25).

Several alternative therapeutic approaches to target early and intermediate-stage retinal degeneration, including gene therapies to restore normal cell function, neuroprotection to support cell survival and reduce inflammatory signalling, pharmacotherapies to compensate for metabolic and biochemical imbalances (26), exosome therapies to deliver packages of mRNAs, miRNAs, proteins to augment gene expression and cell function (27) and optogenetics to confer light sensitivity on other retinal cells (28). Luxturna is the first and only FDA-approved gene therapy for the treatment of an IRD (LCA), however, other disease targets are under development, including AMD and RP using gene-specific and gene-indifferent approaches (16, 29, 30). The long-term efficacy of these therapies is yet to be determined in patients.

Human photoreceptors do not have the capacity to regenerate, and there are currently no treatments to restore vision once they are lost. Cell replacement is a promising approach to treat various types of retinal degeneration. Human pluripotent stem cells offer the potential for unlimited amounts of transplantable retinal cells and photoreceptors. This review discusses progress and challenges in the efficient production of photoreceptors from human pluripotent stem cells and their clinical translation.

### 1.1 Cell transplantation for retinal degenerative diseases

The purpose and goal of cell therapy for retinal diseases is to restore or improve vision in individuals who suffer from various retinal disorders. In the early stages of age-related macular degeneration (AMD), cell therapies aim to preserve cone photoreceptors by transplanting retinal pigment epithelial (RPE) cells (31). In advanced stages of AMD, transplantation of both RPE and cones may be required to restore vision, while other retinal degenerative diseases may require the transplantation of rods and/or cones.

The possibility of photoreceptor integration depends on the structural and functional preservation of the remaining retina, particularly the ONL and retinal ganglia (32, 33) to process and transmit visual information to the brain (34). To achieve retinal integration, donor cells are transplanted into the subretinal space above the RPE layer and must then migrate and extend processes towards the host ONL while overcoming the barriers of the outer limiting membrane (OLM) (35). Subsequently, photoreceptors must mature and function normally through interactions within the surrounding retinal environment.

The retina is an ideal anatomical location for cell transplantation as it is relatively simple to access and monitor and requires relatively few cells to achieve a therapeutic effect (36). Furthermore, the eye is an immune-privileged site with a reduced risk of graft rejection (37). Graft function can be assessed using several non-invasive techniques, such as optical coherence tomography, fundus autofluorescence, fluorescein angiography and visual field tests (38, 39). In the past four decades, the potential for cell therapies to treat various retinal conditions has spurred the development of surgical techniques and has led to numerous clinical trials (40–45).

The transplantation of cells into the retina to treat degenerative diseases has been pursued since the early 1980s. Proof-of-concept was first established in owl monkeys by Peter Gouras and others, who subsequently published a series of studies demonstrating photoreceptor rescue by RPE transplantation in rodents (46). Photoreceptor transplantation was pioneered using retinal microaggregates and enriched photoreceptors in rodents and pigs (47–51). Studies focused on the fundamentals of surgery, preparation of cells and tissues for grafting, and labelling methods to track grafted cells *in vivo*. The feasibility and short-term efficacy of retinal tissue grafting were tested by transplanting retinal clumps from newborn rats into the subretinal space of adult rats with outer retinal lesions (48, 52–55). Studies in wild-type and degenerative model rodents demonstrated graft survival for up to nine months, and preliminary evidence of integration, suggested by the formation of primitive outer segments and synapses (48, 56, 57), and in some cases demonstrated observable behavioral changes such as light avoidance in rodents (58).

Transplantation of retinal tissues and cells of various developmental ages suggested that younger cells provided better outcomes than more developed cells (59). In 2006, Maclaren determined that the optimal developmental age of photoreceptors in mice was post-mitotic precursors, derived from postnatal days 3 and 7 in mice (60). This preference may be attributed to the enhanced resilience exhibited by these precursors relative to fully matured cells, characterized by elongated and delicate structures that may render them vulnerable to dissociation techniques. Additionally, precursors may be better suited to migrate from the subretinal space to establish synaptic connections with the ONL.

Preclinical studies in large animal studies showed subretinally transplanted retinal sheets survived and improved cone responses, despite a lack of evidence of retinal integration (51, 61). However, several human trials conducted between the late 1990s and early 2000s using fetal retinal sheets and cells resulted in limited or transient visual benefit in RP patients (62–65).

Despite their lack of clinical efficacy, these pioneering studies demonstrated that photoreceptor replacement could potentially be developed into a therapy for retinal degenerative diseases by overcoming the obstacles of survival and retinal integration, as well as a reliable source of cells.

### 1.2. Human pluripotent stem cells in cell therapy

The isolation of human embryonic stem cells (hESCs) in 1998 (66) and the subsequent generation of human induced pluripotent stem cells (hIPSCs) by somatic cell reprogramming in 2007 (67, 68) opened a multitude of opportunities for regenerative medicine.

The critical properties of hESCs and hIPSCs (together, hPSCs) include pluripotent differentiation potential (the ability to form any cell type of the body) and self-renewal (maintenance of an undifferentiated state during mitosis), making them an ideal and possibly limitless source of cells for therapeutic purposes. Unlike hESCs, hIPSCs do not require human embryos and can be generated by reprogramming somatic cells. Reprogramming allows for the generation of hIPSCs with patient-specific genetics for personalized medicine and disease modelling and avoids the ethical concerns associated with human embryo research (69).

One of the most promising applications of hPSCs is cell replacement therapies for retinal degenerative diseases. The earliest completed human trials to use hPSC-derived cell products were hESC and hIPSC-derived RPE cells in AMD patients (70, 71). Over the past decade, hPSC-RPE transplantation accounted more human trials than any other hPSC-derived product (22/102) (72). Moreover, the first clinical trial for RP using hIPSC-derived retinal organoid sheets (jRCTa050200020) recently began in Japan (73).

Most clinical trials for AMD have generated hPSC-RPE by spontaneous differentiation (70, 74–76). This approach is inefficient, time-consuming and highly variable across cell lines (77). Furthermore, hPSC-RPE cell stocks must be expanded to generate sufficient cell numbers, leading to reduced cell function and increased risk of mutations (78). By contrast, the directed differentiation of hPSC-RPE cells using cytokines and small molecules results in higher efficiencies and final yields (79–81). Some groups have refined their protocols to produce hPSC-RPE under current good manufacturing practice (cGMP) conditions for human trials (70, 75, 82–84), and commercial approval, though not yet granted, is anticipated (44).

Although photoreceptors and RPE cells are derived from the same progenitor cell type, photoreceptors are more challenging to generate efficiently and are post-mitotic. These two factors make producing large numbers of cells difficult for clinical use. It is, therefore, critical that efficient methods for the production and harvest of photoreceptors can be developed for therapeutic applications (85).

### 1.3. Retinal development *in vivo*

Developing differentiation protocols for specific retinal cell types requires knowledge of retinal development *in vivo*. While the key drivers of early retinal development are known, the extrinsic factors that drive the development of specific neuronal cell subtypes are not yet fully understood.

**Figure 2:**
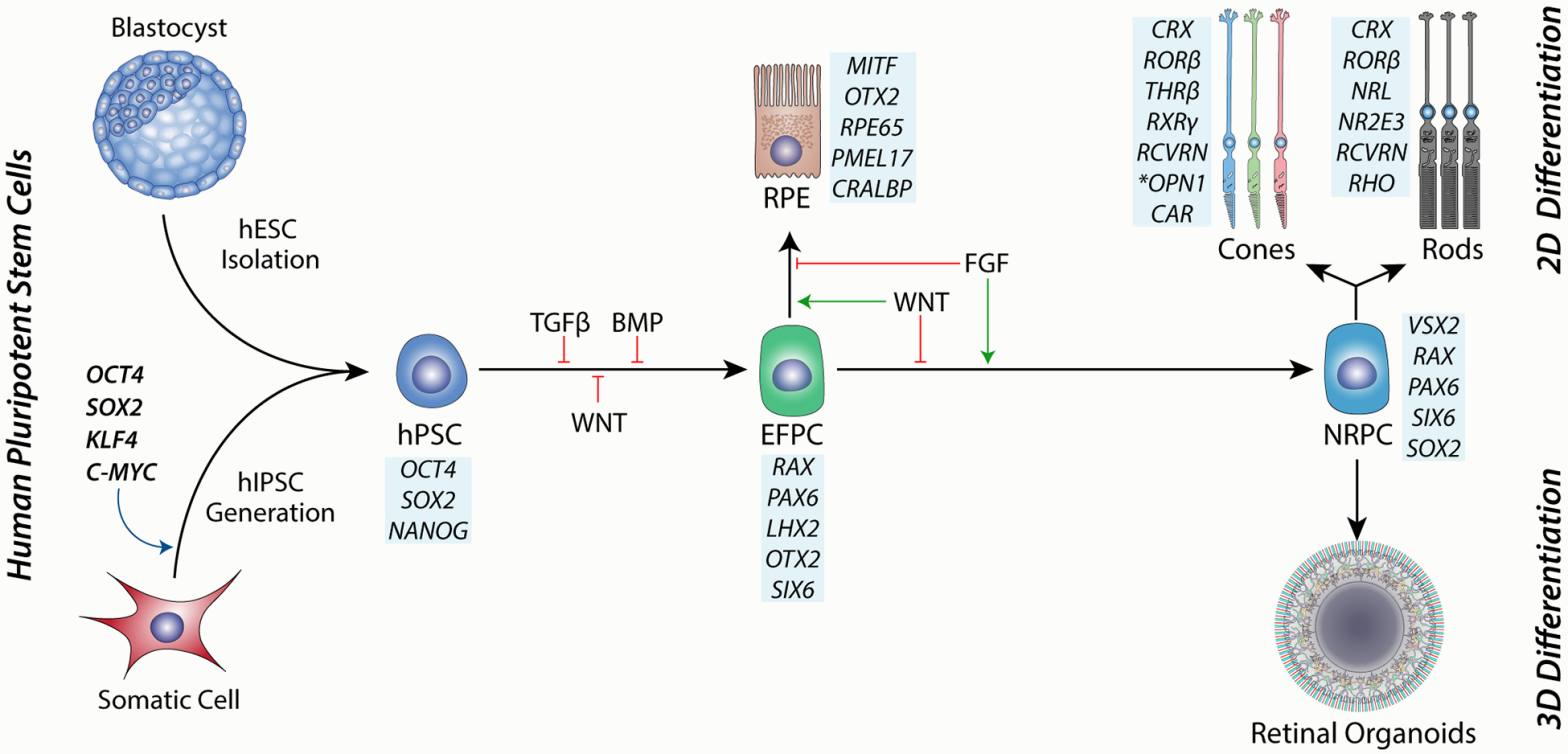
A schematic showing the source of human pluripotent stem cells from either a blastocyst-stage embryo or through hIPSC cell induction by reprogramming factors. hPSCs are then directed toward the eye field progenitor cells (EFPCs) by inhibition of TGFβ, BMP and WNT signalling Progression of differentiation to the RPE is governed by WNT signalling and is enhanced through inhibition of FGF signalling. Conversely, the differentiation of EFPCs towards neural retinal progenitor cells (NRPCs) is driven by FGF signalling and inhibition of WNT signalling. NRPCs have the differentiation potential to form all cells of the neural retina and can be cultured in suspension as 3D aggregates to form retinal organoids or under 2D conditions under directed differentiation towards rod or cone photoreceptors. Blue boxes show markers for each cell type.

Ontologically, the retina is part of the brain and follows early forebrain development *in vivo*. During mammalian embryogenesis, this is achieved by dual SMAD inhibition of TGFβ and BMP signalling pathways, as well as inhibition of WNT signalling along an anterior-posterior gradient that defines this axis, resulting in the anterior neurectoderm (Figure 2) (86, 87). hPSCs can be directed to the same fate using small molecules or cytokine inhibition of the same pathways (81, 88, 89). Eyefield progenitor cells (EFPC) are multipotent and have the potential to form the RPE, neural retina and associated progeny cells, including photoreceptors. Under appropriate conditions, EFPCs can be generated with nearly 100% efficiency, as determined by the expression of specific eyefield markers like RAX, LHX2 and PAX6 (90). The efficient formation of hPSC-derived EFPCs *in vitro* is thus a critical platform for producing the RPE or neural retinal progenitor cells.

Bifurcation of the eyefield to the RPE or neural retina is governed by the expression of MITF and VSX2, respectively (91). Extrinsic factors that regulate this process include FGF and WNT signalling (92). Support for this model can be observed *in vitro* and *in vivo*, where FGF signalling is required for the development of the neural retina in the developing chick (93) and is sufficient for the transdifferentiation of the RPE to the neural retinal *in vitro* (94). This model complements studies showing that loss of WNT signalling during mouse development results in the transdifferentiation of the presumptive RPE to the neural retina (95) and is also supported by retinal differentiation studies using hPSCs (91). Thus, the directed differentiation of EFPCs to neural retinal progenitor cells (NRPCs) has the potential to yield photoreceptors with high efficiency *in vitro*.

### 1.4. Towards photoreceptor production

Early studies on the directed differentiation of mouse embryonic stem cells (mESCs) and hPSCs towards retinal cells used a combination of 3D/2D hybrid protocols to achieve retinal differentiation and, in some cases, showed the production of photoreceptors after extended culture periods. In 2005, a landmark study demonstrated the generation of retinal precursors by culturing mESCs as embryoid bodies in serum-free conditions in the presence of Dkk1 and LeftyA, followed by the addition of serum and Activin, resulting in about 6% Rx+/Pax6+ cells (96). Overexpression of Crx in these cells through lentiviral transduction, or co-culture with retinal explants, resulted in the upregulation of rhodopsin (Rho) and recoverin (Rcvrn).

Shortly after, Lamba et al. (97) demonstrated retinal progenitor differentiation by culturing hESCs as embryoid bodies in serum-free media (SFEB) with IGF-1, DKK1 and Noggin. After three weeks, cells expressed PAX6 and VSX2 (CHX10) in approximately 80% of cells (97). In 2008, Osakada et al. adapted and refined SFEB differentiation in mouse, monkey and human ESCs using retinoic acid and taurine to enhance rod production by day 130 (98). This study also marked the first-time photoreceptor progenitors were generated in the absence of mature retinal tissue, with photoreceptors representing about 20% of cultured cells.

Lamba et al. (99) also demonstrated the differentiation of multiple hESC and hIPSC lines towards retinal progenitors through the suspension culture of aggregates in DKK-1, IGF-1 and Noggin. These cultures showed patches of retinal pigment epithelial, amacrine and neural ganglion cells. After two months of culture, photoreceptor markers, including Crx and Otx2, were detected in about 10% of cells. Approximately 30% of the photoreceptors produced were rods, as indicated by the expression of Nrl, and about 1% of cells expressed more mature photoreceptor markers such as recoverin, AIPL-1, Rho and S-Opsin (99).

By contrast, Meyer et al. (100) induced retinal differentiation using a 3D/2D/3D protocol by culturing hIPSCs without specific factors. For this, hIPSCs were cultured in a neural induction medium (N2 and heparin) from day 2 to day 16, followed by a retinal induction medium (B27) thereafter. Floating aggregates were plated to form neural rosettes, which were lifted to form neurospheres and, over time, proceeded towards photoreceptors via a neural retinal intermediate stage. Despite the absence of specific factors, some cells expressed PAX6 and RAX at day 10, CHX10 by day 40, followed by cells expressing CRX, RCVRN and OPSIN by day 80 (100).

These early studies demonstrated the potential for generating retinal tissue from hPSCs by directed (guided) or unguided methods and laid the foundations for new methods to generate retinal cells and photoreceptors.

### 1.5. Retinal organoid differentiation

The Sasai group advanced retinal differentiation through the generation of 3D self-folding optic cups from mouse (101) and human ESCs (102), leading to the field of retinal organoid research.

Retinal organoids are 3D multi-layered structures derived from hPSCs that can faithfully recapitulate key elements of retinal development, disease and function in the dish (103). Mature retinal organoids contain photoreceptors, bipolar, horizontal, amacrine, retinal muller and ganglion cells and exhibit aspects of native connectivity, apical and basal polarity, cellular architecture, and metabolic interactions (104) and, in some cases, are light responsive (105).

Retinal organoids have since emerged as valuable tools for the investigation of retinal development (106) and diseases *in vitro* (107), therapeutic drug screening (108), exploration of gene therapies (109), and evaluation of neuroprotective agents (110). Furthermore, retinal organoid technologies have reached human trials to treat RP (111).

Over the past decade, many research groups have adapted and explored new protocols for retinal organoid generation. Here, we discuss the common features of retinal organoid protocols and highlight fundamental research that has improved organoid development's robustness and quality and increased photoreceptor yields and quality.

**Figure 3:**
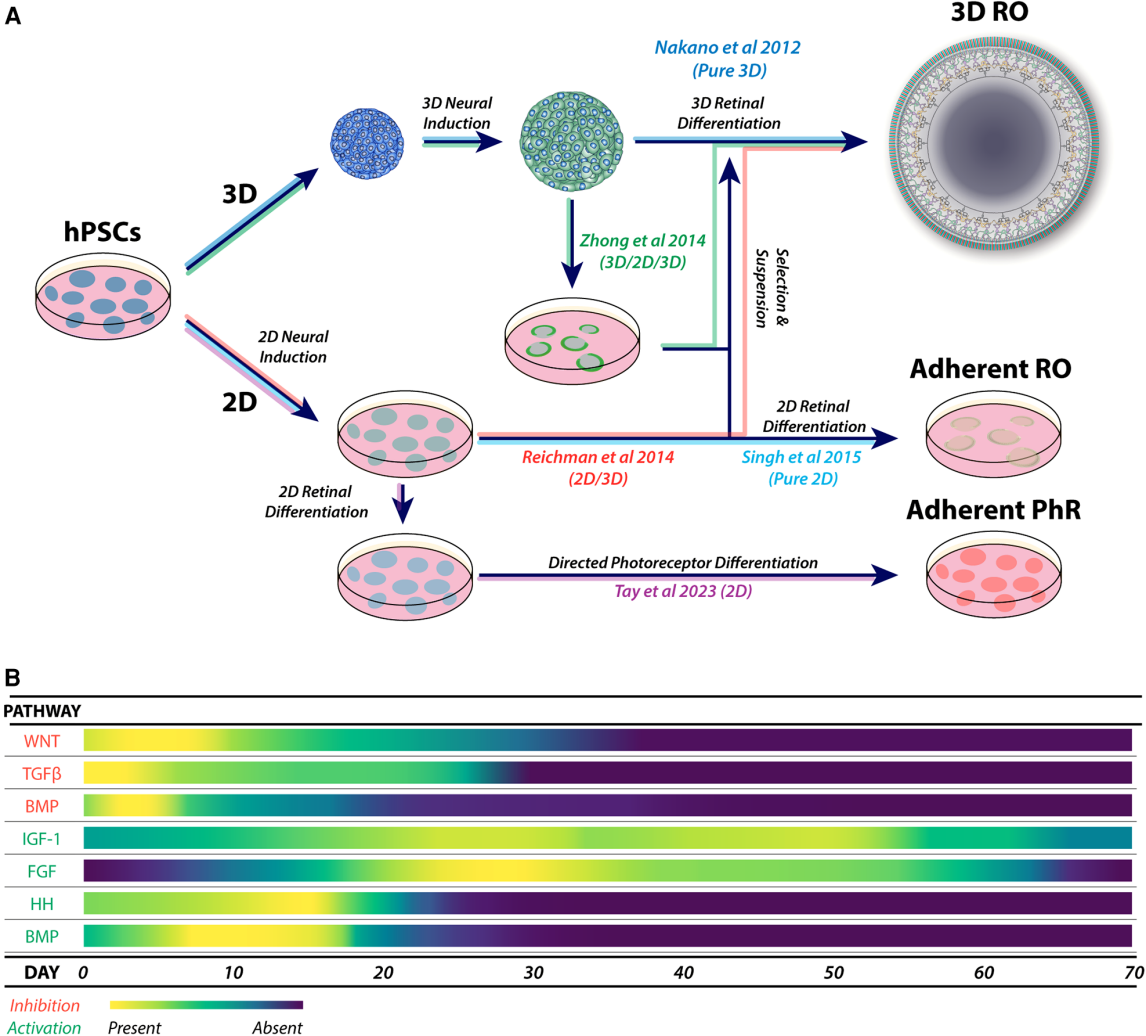
(**A**) Major variations in the physical formats for retinal organoid and photoreceptor differentiation, showing combinations of 3D, 2D/3D, 3D/2D/3D and 2D culture systems. These approaches form the basis of derived and adapted protocols for the generation of retinal organoids and enhanced photoreceptor differentiation. (**B**) A heat map of the most utilized pathways during guided neural induction and early-to-mid retinal differentiation. Pathway activation or inhibition is commonly achieved using small molecules and exogenous cytokines. Adaptations of protocols are commonly explored by using additional specific molecules to enhance or promote target cell types, including photoreceptors.

Retinal organoid protocols generally share features of neural induction, isolation or selection of developing neural retinal tissue, followed by culture to support retinal development. Neural induction can be performed using three-dimensional (3D) (112) or two-dimensional methods (2D) (113) (Figure 3A) and may be directed (guided) using specific signalling factors or unguided (spontaneous) (100, 114, 115), which is consistent with the default model of development (116). While unguided protocols may be sufficient for some cell lines, inherent differences between lines, such as epigenetics (117, 118), may affect retinal differentiation (99, 119, 120).

Guided protocols provide a form of controlled neural induction that may override the intrinsic bias of hPSC lines (121). Guided neural induction typically involves inhibiting the TGFβ, BMP (122–126) and WNT signalling pathways to enhance anterior neural differentiation (123, 127–129) (Figure 3B). Recently, nicotinamide was shown to enhance neural induction through BMP inhibition (130) and possibly also through inhibition of the WNT pathway (131).

Retinal differentiation may be enhanced by promoting neural retinal differentiation and development and inhibiting RPE differentiation. A common approach is stimulation of the FGF pathway using FGF2 (132–133) or FGF9 (94, 134, 135). Other approaches include the direct inhibition of WNT signalling with small molecules or factors (102, 125, 134, 136).

Supplementation of retinal organoid cultures with IGF-1 promotes optic vesicle and optic cup formation, retinal lamination and the appearance of photoreceptors (112, 137). Various protocols employ BMP4 soon after neural induction to enhance retinal differentiation (138–140). However, the effects of IGF-1 and BMP4 on retinal organoid differentiation may be protocol and cell line specific (141). Despite these observations, retinal induction with BMP4 is reproducible across multiple hPSC lines (73, 139, 142, 143).

Amongst the myriad of retinal organoid protocols, a unique approach to neural retinal development by Kuwahara et al. (138) used the directed differentiation of hPSCs to RPE cells followed by induction reversal to the form highly organized organoids with a stratified neural retina that were rich in photoreceptors (138). This protocol has been further optimized by preconditioning hPSCs through SHH activation and TGFβ inhibition in stem cell maintenance medium for 24 h (142).

### 1.6. Photoreceptors from retinal organoids

Strategies for promoting photoreceptor differentiation in retinal organoids vary from improving overall organoid quality, increasing photoreceptor yields, biasing photoreceptor sub-types and improving final photoreceptor maturity and function.

Notch inhibition causes retinal progenitors to exit the cell cycle, which triggers the early appearance of photoreceptors and increases yields (127, 129, 133). While most studies have used DAPT to inhibit Notch signalling, one study demonstrated that Notch inhibition for three days using PF-03084014, but not DAPT, at different time points (day 45 or day 60 and day 90) promoted differentiation of cones and rods, respectively (144). However, neither cone nor rod photoreceptors did not express mature markers, even after prolonged culture. However, Zerti et al. (145) found that exposure of retinal organoids to DAPT from days 28–42 and retinoic acid (RA) from day 30 to day 120 significantly increased M/l cone differentiation at the expense of rods (145).

Although RA is commonly used to enhance photoreceptor development (114, 127, 146, 147), the timing and concentration of RA can affect the timing of photoreceptors, subtype bias, and maturation (145, 148, 149). Moreover, the type of retinoid can also affect photoreceptor differentiation. Zerti et al. (145) found that RA enhanced expression of mature rod and cone markers, while 9-cis-retinal and 11-cis-retinal reduced expression below control values. Conversely, the Swaroop lab found 9-cis-retinal accelerated rod differentiation (150, 151).

COCO simultaneously inhibits the TGFβ, BMP and WNT (152) signalling pathways and enhances cone photoreceptor differentiation. In an adapted protocol, Pan et al. (125) showed that COCO significantly increased photoreceptor precursors by day 45 but not final photoreceptor yields. However, COCO increased the ratio of cones vs. rods by day 90 (125).

Other studies have used thyroid hormone to bias photoreceptor sub-types. Thyroid hormone determines the development of rod vs. cone subtypes when used in a temporal and concentration-dependent manner and causes a shift away from S-Opsin cones towards M/l-Opsin cones (128, 153, 154). Finally, somatostatin signalling was recently found to promote rod differentiation and maturation (155).

In addition to culture media and signalling molecules, physical conditions can enhance photoreceptor development in organoids. For example, Völkner et al. (156) utilized a combination of hypoxic culture and tri-sectioning, which led to the development of stratified retinal organoids (ROs) expressing markers related to phototransduction and synaptic vesicle proteins, along with functional responses to light (156). Furthermore, improved RO development can be achieved by cultivating them on plate shakers or in spinner flasks (157) and using rotating vessels (158). These approaches facilitate the efficient exchange of oxygen and nutrients, enhancing retinal development.

Overall, the discovery of retinal organoids has led to various methods to create retinal tissues containing photoreceptors of different stages of development, with increasing control over rod and cone differentiation and higher yields that will hasten their clinical translation.

### 1.7. Directed differentiation of photoreceptors

Whereas retinal organoids are intended to recapitulate complexity of the developing retina, directed differentiation protocols aim to generate photoreceptor precursors with high efficiency and may be useful for large-scale production.

Following classical dual SMAD and WNT inhibition using COCO in 3D/2D culture, Zhou et al. (88) generated photoreceptor precursors for 21 days at high efficiency. Neural induction was performed using EBs by using COCO, FGF2 and IGF-1, which were plated onto Matrigel and cultured for a further four weeks, resulting in approximately 90% CRX+ and 40% S-Opsin+ cells by day 21. The addition of thyroid hormone shifted cone identity from S-Opsin to M/l-Opsin cones (88). The remarkable ability of COCO to direct cone differentiation has since been tested in rat retinal progenitor cells (159) and human retinal organoids (125, 126).

Tay et al. (160) explored the effect of culture substrate on photoreceptor differentiation efficiencies (160). hESC monolayers were seeded onto LN521, LN323 + LN521 (2:1 ratio) or LN523 + LN521 (2:1 ratio) and cultured in neural induction medium (N2, B27, SB431542, and CKI-7) until day 9, followed by photoreceptor differentiation medium containing (N2, B27, BDNF, CNTF, RA, and DAPT) until day 32. By day 32, approximately 35% of cells on LN523 + LN521 co-expressed CRX and RCVRN, compared to -10% on LN323 + LN521 and >1% on LN521 alone. This is consistent with the presence of laminin *γ*3 in the interphotoreceptor matrix (161–163).

Direct conversion by transcription factor overexpression presents new ways to generate target cell types from hPSCs (164). This strategy generated photoreceptors from hPSCs by inducible expression of CRX and NEUROD1 under 2D and 3D (165). By day 28, RCVRN was expressed in approximately 34% and 44% of cells in 2D and 3D cultures, respectively. Moreover, RHO and CAR were elevated in 3D compared to 2D culture. While the future clinical application of genetically modified cells remains uncertain, this may be overcome by the direct conversion of hPSCs to photoreceptors using non-integrative techniques, such as mRNA transfection (166).

### 1.8. Co-culture methods

The optic cup stage of retinogenesis involves interactions between the nascent neural retinal layer and the RPE that promote their mutual development and maturation *in vivo* (167, 168). Co-culture systems partially recapitulate this by bringing retinal tissue or photoreceptors into proximity with the RPE, facilitating dynamic signalling between cells *in vitro*.

The RPE is a central source of factors involved in the development, maturation, function and maintenance of photoreceptors, including IGF-1, FGF, CNTF, TGFα, CNTF, PEDF, BDNF and HB-EGF (169). While RPE-conditioned medium can enhance retinal cell differentiation and survival (170–173), co-culture systems restore cell-to-cell contact to potentially enhance differentiation and maturation of retinal cells and the RPE (174–177).

Various combinations of RPE cells, retinal explant sheets, neurospheres, isolated cells, stem cell-derived retinal cells, retinal organoids and retinal cell lines are used in co-culture systems (178). Additionally, co-culture systems may combine cells from different species (179).

Basic co-culture systems typically use RPE monolayers on dishes or transwell systems layered with dissociated retinal cells, resulting in higher maturation than mono-culture controls (180). Studies using hPSC-derived RPE/neuroretinal co-cultures demonstrate enhanced photoreceptor differentiation. hESC-derived 3D neuroretinal progenitors cultured on primary rabbit RPE monolayers led to contact-dependent photoreceptor maturation (181). A proximity effect of RPE and retinal co-culture was shown using RPE cells and retinal progenitors derived from hESCs, resulting in photoreceptor differentiation near the RPE and upregulation of ganglion cell markers on the opposite side, as seen during retinal development (134). Similarly, hIPSC-derived retinal progenitor cells seeded on a scaffold (GCH-521: gelatin, chondroitin sulphate, hyaluronic acid and laminin 521) in contact with mature human fetal RPE monolayers developed a laminar structure with immature photoreceptors and immature inner nuclear layer, as well as increased RPE maturation (182). Subsequent research by the same group revealed that this co-culture system led to the deposition of drusen-like mounds by the RPE, offering a model of early AMD (183).

The co-culture of retinal organoids on RPE cells enables interaction between committed photoreceptor precursors rather than the nascent neural retina and can accelerate the upregulation of mature photoreceptor markers (184). Achberger et al. (178) co-cultured retinal organoids and RPE cells using a microfluidic device, generating mature photoreceptors and recapitulating native interactions, including outer-segment phagocytosis (178).

Thus, co-culture systems demonstrate the potential for RPE cells to improve retinal development and enhance photoreceptor differentiation by reconstructing the native configuration of the developing retina *in vitro* and may also improve therapeutic efficacy when co-transplanted (135, 185, 186).

## 2. Challenges for the clinical use of hPSC-derived photoreceptors

Photoreceptor therapies face several hurdles before reaching the clinic. One of the most important is the accurate determination and optimisation of structural integration into the retina. Even without integration, therapeutic benefit can occur through indirect mechanisms such as the release of supportive factors. Since results from preclinical studies support the clinical translation, the degree, contribution and duration of such mechanisms to therapeutic outcomes should be understood so they can potentially be overcome or exploited (187).

### 2.1. Cell tracking

Early studies labelled donor cell nuclei with triturated thymidine (57) or genetically modified *β*-galactosidase reporters (56). However, thymidine labelling does not provide structural information and can create false positives through host photoreceptor uptake of labelled DNA from dead donor cells (188), while *β*-galactosidase/Lac Z + can be taken up by host cells. It was, however, recognized that dual cytoplasmic and nuclear labelling might be required to interpret transplantation outcomes accurately.

The advent of fluorescent reporters gave rise to new methods for cell tracing and allowed for cell sorting prior to transplantation. Swaroop and MacLaren developed the NRL-GFP model in 2006 for rod transplantation studies (60, 189). Many groups have created photoreceptor reporter lines or used viral delivery to identify and isolate photoreceptors by FACS and follow cell survival and integration in transplanted animals. Reporter lines have also enabled visualization of photoreceptors during *in vitro* differentiation, commonly using CRX, RCVRN, Opsin (102, 190–197),. Alternatively, cell cultures can be transduced with viral reporters to label and isolate specific cells and track them post-transplantation (191, 198–201).

Several groups have transplanted fluorescently labelled photoreceptors into animal models and reported integration based on its co-localisation with synaptic markers or presence in the ONL (99, 153, 194, 198, 200, 202–208). While photoreceptor integration rates are typically below 1%, some studies have also shown significant improvements retinal electrophysiology and animal behaviour (61, 73, 200, 203, 207, 209–211), suggesting the possibility of therapeutic benefit.

### 2.2. Cytoplasmic transfer

Prior evidence for photoreceptor integration was called into question in 2016 and 2017 after a series of studies showed that male GFP+ photoreceptors transplanted into female DsRed mice resulted in a high proportion of cells that were GFP+/DsRed+, suggesting that donor and host photoreceptors shared cytoplasmic contents (190, 212–214). Further analysis showed that cytoplasmic exchange was bidirectional between host and donor cells and mediated through direct contact. However, cell fusion was ruled out by the absence of binuclear cells or fused membranes. The uptake of GFP from the extracellular space was also ruled out by the injection of GFP into the subretinal space (212). Subsequent studies confirmed that material exchange between photoreceptors occurred *in vitro* and *in vivo* post-transplantation via photoreceptor nanotubes rather than EV release (215, 216). Exchanged material between donor and host photoreceptors included miRNA, mRNA, proteins and mitochondria.

The Pearson group demonstrated that rates of donor cell integration and cytoplasmic transfer were affected by donor age and the host background using GFP+ mouse cone precursors from male mice or mESC-derived retinal organoids (217). Male GFP+ cone precursors were transplanted into adult wild-type (WT) and retinal degenerative backgrounds expressing dsRed. Consistent with previous studies, they showed that the developmental stage of donor cells affected integration into wild-type retinas. Specifically, GFP+ cone precursors harvested at E15 integrated less effectively than their counterparts from P1, P8 and mESC-derived ROs. The apparent integration rates of photoreceptors varied significantly across genetic backgrounds, with very few GFP+ cells found in the WT retina and almost 10-fold higher numbers in the NRL-/- retina, compared with other models, suggesting a role of the host's outer limiting membrane (OLM) on transplantation outcomes. Using FACS and fluorescent *in situ* hybridization (FISH), they found that up to 99% of GFP+ cells in the WT retinas were of host origin, compared to approximately 75% in NRL-/- retinas. Taken together, these experiments showed that rates of integration and cytoplasmic exchange can co-occur and are directly affected by the developmental age of donor cells and the host retinal environment, particularly the integrity of the OLM. Finally, cytoplasmic exchange between photoreceptors via nanotubes has been shown to occur naturally in the adult mouse retina and may be a natural homeostatic process (215, 218).

Cytoplasmic exchange via nanotubes between transplanted human and host animals remains underexplored. However, tunneling nanotubes have been known to mediate transfer between various human cells *in vitro* (219). Moreover, such exchanges have also been observed between transplanted hPSC-derived MSCs into mouse cells (220). Thus, it is possible that nanotube-mediated cytoplasmic exchange may also occur in pre-clinical studies of hPSC-photoreceptor transplantation.

The apparent setback of cytoplasmic exchange may also be an opportunity to develop new therapies, including mitochondrial delivery via cell transplantation (221, 222). By mapping the endogenous networks of mitochondrial transfer within the retina, stem cell-derived products could be utilized as mitochondrial delivery vectors to specific retinal cell types. Of particular interest will be rare diseases caused by mutations in the mitochondrial genome (mtDNA) (223). Another application is the delivery of engineered mitochondria (224, 225) to increase the survival of RPE, photoreceptors and other retinal cells with compromised capacity for redox homeostasis (226, 227).

### 2.3. Photoreceptor isolation

Photoreceptor isolation from heterogeneous cultures is challenging, especially for clinical applications. Currently, two main approaches are used for photoreceptor isolation: fluorescent reporter-based fluorescence-activated cell sorting (FACS) or antibody labelling followed by FACS or magnetic-activated cell sorting (MACS) (228, 229).

Fluorescent reporters, under photoreceptor-specific gene regulation, enable isolation from heterogeneous cultures, facilitating quantification of differentiation outcomes, characterization, animal transplantation, real-time survival monitoring, and retinal integration studies (102, 153, 192, 196, 197, 200, 202). However, their clinical use is limited due to potential risks from genetic manipulation and fluorophores' cytotoxic and immunogenic effects (230). Hence, it is crucial to isolate photoreceptors based on native characteristics, like surface markers.

The surface marker CD73 was initially considered a promising target for photoreceptor isolation (231). CD73+ selection of day 120 ROs resulted in approximately two-fold enrichment of CRX+/RCVRN+ photoreceptor precursors, capable of producing cones and rods *in vitro* and *in vivo* (229). However, different studies' attempts to isolate photoreceptors from ROs demonstrated vastly different results.

Lakowski et al. (232) found few CD73+ cells in day 100 ROs and human fetal retinas. Instead, CD73 was highly prominent in day 200 ROs and human adult retinas. Unexpectedly, CD73+ sorting of day 200 ROs resulted in an approximately six-fold depletion of CRX+/RCVRN+ photoreceptor precursors from the total RO cell pool, suggesting that CD73 is a poor marker of early photoreceptor differentiation dynamic changes in the co-expression of CD73, CRX and RCVRN during early development and poor cell type specificity (232). A similar study sorted day 150 ROs using RCVRN-eGFP reporter and found almost no detectable transcript for NT5E (gene coding for CD73) and poor CD73 co-localization with eGFP+ cells by immunofluorescence. Rather, eGFP expression was colocalized with rod and cone photoreceptor markers and CD133 (196).

In a concerted effort to establish a robust and reliable photoreceptor marker panel, Welby et al. (192) conducted a high throughput study of 242 markers on developing human foetal retinas transduced with a cone-GFP reporter. The screen identified several candidate markers but none that were unique to cones. Candidate markers were tested against cone-GFP transduced ROs (∼day 120), resulting in a sorting strategy (SSEA1-/CD26+/CD133+/CD147+) with approximately seven-fold enrichment. However, only 30% ± 15% of the isolated cells expressed cone markers (192).

These studies underscore the complexity of photoreceptor isolation using antibody-based methods, especially for clinical applications. In addition to exploring high-yielding marker panels, novel purification methods like label-free isolation techniques may also prove useful (233–235).

### 2.4. Further considerations for transplantation

The outer limiting membrane is a physical barrier to photoreceptor integration—studies using models of photoreceptors integrated at far higher efficiencies when coupled with OLM disruption. Pearson et al. (212) showed that approximately 23% of photoreceptors integrated into such models, compared with models with an intact OLM. This is consistent with previous research showing enhanced integration through transient chemical disruption of the OLM (236) or through genetic disruption (237). However, the severity of OLM disruption is also linked to disease progression, which may hinder integration due to retinal remodelling, glial scarring, and inflammation (239, 240). In the context of cell therapy, this would imply an optimal therapeutic window, or pre-treatment of the retina to limit retinal remodelling and gliosis.

Strategies for clinical therapies must reflect disease progression and the state of patient retinas. For instance, the transplantation of hPSC-RPE cells may be sufficient for AMD patients with viable cones; however, advanced stages of AMD will likely require co-transplantation of both hPSC-derived RPE cells and cones prior to retinal remodelling (238). On the other hand, low photoreceptor integration rates and widespread degeneration limit the potential of recovery of full-field vision rescue in RP patients. However, an appropriate strategy might be to transplant rods around the macula to protect cones and save central vision. This approach would not necessarily depend on the integration and maturation of rod cells for light detection but would instead rely on the neurotropic effect of rod-derived cone viability factor (RdCVF) to enhance the survival of cones via increased glucose uptake (10). Proof of concept for this approach has been demonstrated in a porcine model of retinitis pigmentosa, where photoreceptor precursors from pig embryos and hPSCs prevented the loss of cones up to 1,000 μm from the site of injection and reactivated dormant cones when performed prior to cone inner segment disassembly (239). Thus, therapeutic strategies should be designed in a disease specific manner to achieve the best outcome possible.

The immune privilege of the eye is not absolute in health and is often compromised in the diseased state. Therefore, one major hurdle in the utilization of hPSCs for cell therapies is a requirement for human leukocyte antigen (HLA) matching between the donor cells and the recipient, as mismatched HLA can lead to immune rejection and decreased therapeutic efficacy (240). Using autologous hIPSC lines offer a perfect HLA match for the patient, however, the current technological limitations, including the time and effort required to generate and validate patient-specific hIPSC clones and the need for genetic modification of disease alleles before differentiation, make this approach impractical (84, 243).

A truly universal donor stem cell line would allow off-the-shelf therapies to be available to the entire population. At present, gene editing is used to suppress surface expression of HLA-I and HLA-II proteins, while simultaneously preventing attack by Natural Killer (NK) cells (244). This is primarily achieved in hPSCs by deleting the genes encoding for Beta-2 Microglobulin (*B2M*, encoding the subunit required for surface expression HLA-class I proteins) and *CTIIA*, which encodes a transcription factor necessary for HLA-class II proteins (245). Complimentary or combinatorial approaches have also been used to prevent NK attack, reduce immune co-stimulation or enhance tolerance by overexpression of other molecules like CD47, HLA-E, HLA-G, PD-L1, CTLA4-Ig (246–248).

Alternatively, HLA homozygote superdonor stem cell banks can be generated to match the HLA-A, HLA-B, and HLA-DR loci of populations for transplant compatibility (249). The degree of population compatibility depends on the genetic diversity within a given population. For instance, it is estimated that 30–55 HLA homozygous lines cover 80% of the Japanese population (250), while 150 lines would be needed for 93% compatibility in the UK (251) and 80 lines for 50% compatibility in California (252).

Each approach towards immunocompatible cells is imperfect and comes with its own compromises. For instance, patient specific hIPSC lines are a perfect HLA match to the donor but are expensive and time consuming to create product cells from the initial biopsy. HLA homozygous lines achieve compatibility with limited subsets of any given population without immune evasion or suppression, and thus may may present viral or cancer antigens in a normally. Finally, universal HLA-edited lines may be compatible with the entire population, although there are some safety concerns about compromised antigen presentation and mimicry of cancer survival strategies.

## 3. Conclusions

Advancing cell-based therapies to address retinal degenerative diseases necessitates interdisciplinary progress. Over the past five decades, significant advancements in areas such as transplantation, immunology, molecular, cell biology, and materials science have increased the possibility of cell therapies to treat retinal degeneration—and perhaps restore vision—within our lifetimes. This is particularly evident in the rapid progress in cell production and supportive technologies like GMP facilities and the establishment of hPSC banks.

However, recent insights into cytoplasmic exchange between donor and host photoreceptors reveal that genuine integration rates are notably lower than initially estimated, thereby elevating the evidential threshold for researchers. Such findings underscore the need to revisit critical facets of photoreceptor transplantation, including the optimal differentiation state and delivery modality, be it through injections of singular cells, retinal sheets, or concurrent delivery of RPE with retinal cells. Given the inconsistencies in transplantation outcomes across animal models, novel models may be needed to assess and predict photoreceptor integration in the context of human retinal pathology.

Complementary therapeutic strategies, such as neuroprotection, EV therapies, gene interventions, immune modulation, and optogenetics may reduce the need for cell replacement, delay regeneration or extend the optimal window for cell transplantation. Such approaches may be complementary or necessary to pre-treat the retina before cell transplantation for optimized therapeutic outcomes. Such strategies might be indispensable for pre-treatment before cell transplantation to maximize therapeutic efficacy. Inevitably, human clinical trials will play a pivotal role in establishing human specific approaches and widespread adoption of photoreceptor transplantation therapies.
